# Social motivation is associated with increased weight granted to cooperation-related impressions in face evaluation tasks

**DOI:** 10.1371/journal.pone.0230011

**Published:** 2020-04-20

**Authors:** Lou Safra, Nicolas Baumard, Valentin Wyart, Coralie Chevallier

**Affiliations:** 1 Sciences Po, CEVIPOF, CNRS, UMR7048, Paris, France; 2 Laboratoire de Neurosciences Cognitives et Computationnelles (LNC2), Département d'études cognitives, Inserm, U960, Ecole Normale Supérieure, PSL Research University, Paris, France; 3 Département d'études cognitives, Ecole Normale Supérieure, CNRS, UMR8129, Institut Jean-Nicod, PSL Research University, Paris, France; Institut VEDECOM, FRANCE

## Abstract

It is a trope in psychological science to define the human species as inherently social. Yet, despite its key role in human behaviour, the mechanisms by which social bonding actually shapes social behaviour have not been fully characterized. Across six studies, we show that the motivation for social bonding does not indiscriminately increase individuals’ willingness to approach others but that it is instead associated with specific variations in social evaluations. Studies 1–4 demonstrate that social motivation is associated with a larger importance granted to cooperation-related impressions, i.e. perceived trustworthiness, during social evaluations. Studies 5 and 6 further reveal that this weighting difference leads strongly socially motivated participants to approach more partners that are perceived as both dominant and trustworthy. Taken together, our results provide support for the idea that humans’ social motivation is associated with specific social preferences that could favour successful cooperative interactions and a widening of people’s cooperative circle.

## 1. Introduction

Countless papers state in their opening lines that ‘*humans are a highly social species*’. The ability and motivation to form social bonds plays a key role in human behaviour and has a strong effect on individuals’ fitness [1–3]. However, the mechanisms by which this social tendency shapes social behaviour have yet to be fully characterized. Being ‘motivated’ to engage with others can mean different things. The simplest view is that a higher motivation for social bonding increases individuals’ overall willingness to engage with others; a more nuanced view is that social motivation operates as a targeted strategy that shapes social preferences to maximize cooperative goals. Given that social interactions carry important exploitation risks, we posit that a strategy that would indiscriminately increase people’s willingness to approach others would have detrimental consequences for individuals’ success [4–6]. Our specific hypothesis therefore, is that the motivation for social bonding is shaped in a way that mitigates these risks. Specifically, as a first step to understand the precise effect of social motivation on cooperative behaviour, we hypothesize that the motivation for social bonding is associated with specific differences in social preferences that may ultimately maximize the number of *successful* cooperation interactions by strategically widening individuals’ social circle.

Given that motivation influences social perception [7,8], we test this hypothesis by modelling individuals’ social preferences during face evaluation tasks. More precisely, we measured both the general tendency of socially motivated individuals to rate others as likeable or threatening, as well as the weight they grant to two fundamental social signals: trustworthiness and dominance.

Previous work in social cognition has shown that social evaluations can be decomposed along these two dimensions: trustworthiness evaluations relate more specifically to cooperation while dominance evaluations relate more specifically power or coercion [9–12]. The importance of these two axes varies across social contexts [9–12]. For instance, leader preferences are more driven by dominance than by trustworthiness in war-time, when physical strength is important, but they are more affected by trustworthiness in peace-time [13–15].

In the present studies, we use face evaluation tasks to test the association between people’s motivation for social bonding and differences their social evaluations and preferences. Our prediction is that increased social motivation reflects a cooperative strategy, and should therefore be associated with variations in the weight participants grant to cooperation- and power-related social impressions. This methodology focuses on individuals’ subjective trustworthiness and dominance impressions and is agnostic about the actual validity of these impressions. In other words, the advantage of this method lies in the fact that the weight participants grant to perceived trustworthiness or perceived dominance is an interesting way to reveal their social preferences for trustworthiness and dominance (even if these impressions may not correlate with objective traits). This methodology also allows us to focus on trustworthiness and dominance, which are associated with stable first impressions in facial evaluation tasks, and are important characteristics in social partners: Trustworthy partners, on the one hand, are more likely to cooperate, powerful and dominant partners, on the other hand, are more likely to provide large benefits in the case of a successful cooperative interaction and to impose larger costs in the case of exploitation.

## 2. Study 1 –Threat evaluations

**Fig 1:**
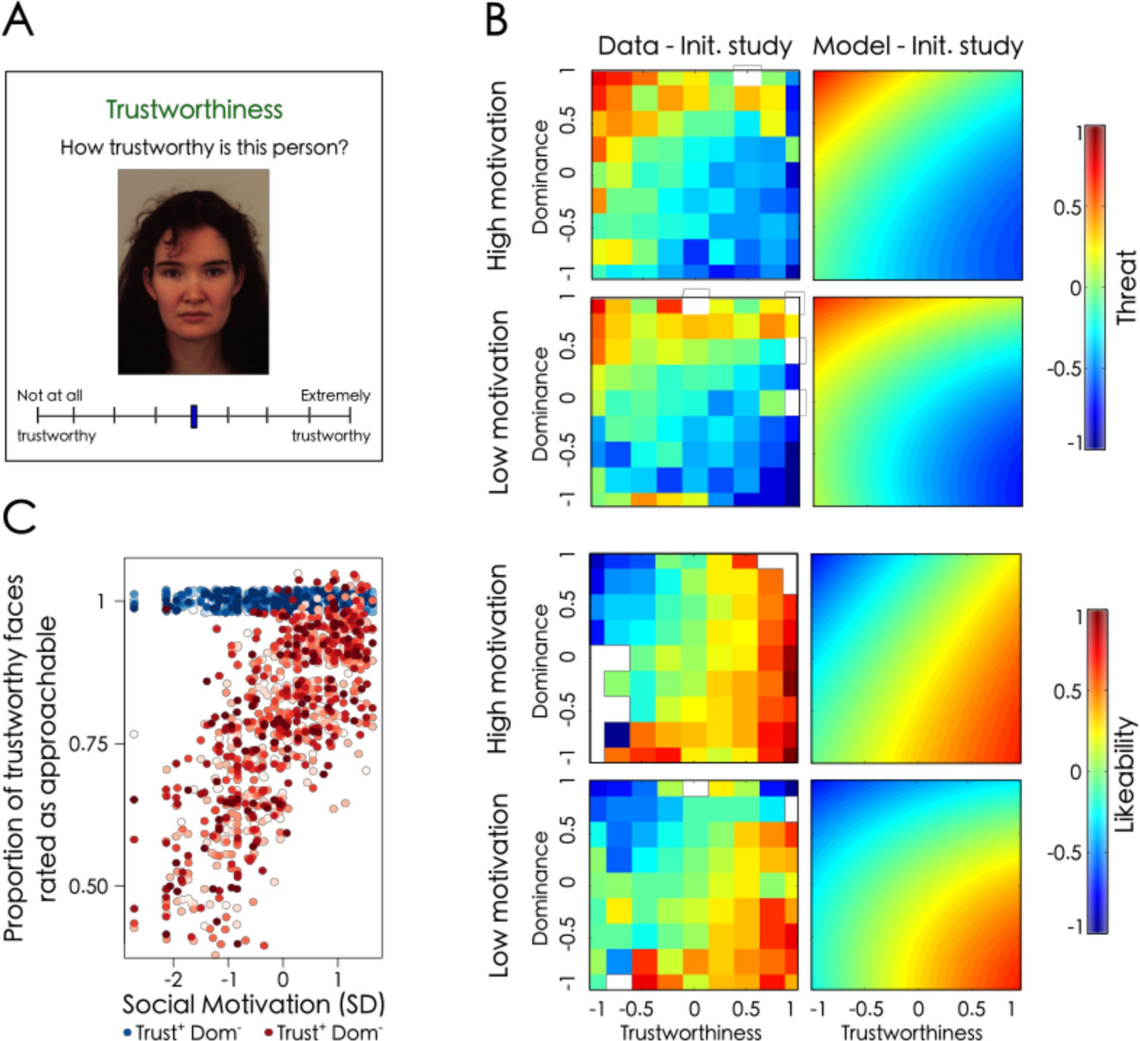
The motivation for social bonding is associated with an increase in the relative importance granted to trustworthiness during social evaluations. (A) Example of an evaluation trial. Participants had to rate each face by moving a cursor, initially positioned in the middle of the scale (Face from the Karolinska database, ID: AF04). (B) Threat (top) and Likeability ratings (bottom) as a function of trustworthiness (x axis) and dominance ratings (y axis). Rating intensity is represented on a scale ranging from blue for lower ratings to red for higher ratings. Pixelized figures correspond to averaged data in the Studies 1 and 4 for the most (upper row) and least (lower row) socially motivated participants (median split). Smoothed figures represent the predictions of the regression models for these studies. Faces perceived as both dominant and trustworthy are rated as more approachable (i.e. likable or not threatening) by participants with high social motivation scores. (C) Effect of social motivation on the evaluation of trustworthy faces. Social motivation is associated with an increase in the proportion of dominant and trustworthy (Trust^+^Dom*, in red) but not of submissive and trustworthy faces (Trust^+^Dom^-^, in blue) perceived as approachable. The different simulations are represented by different shades of blue and red.

To test whether the motivation to form social bonds is associated with a generalized increase in the tendency to rate others as likeable or non-threatening or with a more specific preference for cooperation-related impressions, we first examined participants’ reliance on perceived trustworthiness and dominance to produce threat evaluations. More precisely, we asked 60 participants to rate 40 faces on threat, trustworthiness and dominance (Fig 1A). We then modelled participants’ threat evaluations as a function of perceived dominance and perceived trustworthiness.

### 2.1. Material and methods

#### Ethics statement

All our studies were approved by the local Ethical Committee (Conseil d’évaluation éthique pour les recherches en santé–CERES n°201659) and each participant received a description of the study and signed an informed consent before starting the experiment. All scripts and raw data are available in the OSF project (https://osf.io/x8p4t/).

#### 2.1.1 Participants

Participants recruited via Amazon Mechanical Turk (MTurk, http://www.mturk.com), which offers a large diversity of participants [16,17]. The number of participants was fixed *a priori* based on Chevallier et al. [18], who reported significant correlational effects of social motivation in samples of 26 participants tested in the laboratory. Knowing that online experiments may be more susceptible to noise due to less controlled settings, we decided to test 60 American participants (28 women), aged 23 to 65 years old (34.92± 2.57 years). Each participant received 2$ for completing the task (which corresponds to the prorated average 6$/hour compensation commonly used on MTurk).

#### 2.1.2. Experimental procedure

*2*.*1*.*2*.*1*. *Rating task*. The experiment was programmed on Qualtrics (http://www.qualtrics.com) and lasted approximately 20 minutes. Participants were asked to rate 40 neutral faces (20 woman faces) extracted from the Karolinska database [19] on trustworthiness, dominance and threat. Presenting both male and female faces was used as a mean of having larger variations of perceived dominance and perceived trustworthiness as gender is associated with differences in the evaluations on these two traits [10]. Participants had to answer: “How [trait] is this person?” using a cursor on a 9-point scale ranging from 1 “not at all [trait]” to 9 “extremely [trait]” (recoded from -1 to +1 for the analyses), with [trait] replaced with “trustworthy”, “dominant” or “threatening” (Fig 1A). Following Oosterhof and Todorov’s methodology, the questions bearing on each trait, i.e. trustworthiness, dominance or threat, were presented in different blocks and male and female faces were analyzed together [10].

Participants were instructed to answer following their first impression and they were told that there was no right or wrong answer. The cursor was initially set to the middle in order to reinforce the salience of the positive and negative ends of the scale. If the participant wanted to select the middle of the scale, she still had to click on the scale to generate a response. The name of the dimension was displayed in each trial.

*2*.*1*.*2*.*2*. *Questionnaires*. After the social evaluation task, participants completed the Social Anhedonia Scale [20,21] (*M* = 15.92 ± 2.74, Cronbach's *α* = .85), which is a 40-item self-report questionnaire that has been previously used as a measure of social motivation in various populations, including healthy adult participants [18,22]. The scale includes items such as ‘*Although I enjoy doing by myself I usually seem to have more fun when I do things with other people*’ and ‘*A car ride is much more enjoyable if someone is with me’*. Because they find social interactions more rewarding, individuals low in social anhedonia are more motivated to form social bonds. The entire experiment lasted approximatively 20 minutes.

#### 2.1.3. Data cleaning

The faces were presented simultaneously with a word indicating which trait participants had to rate the face on, e.g., dominance or trustworthiness. Participants had to give their ratings as soon as the face appeared on the screen. In order to ensure that participants had processed the face before placing their cursor on the scale to give their response, reaction times inferior to 200 ms were discarded (percentage of excluded trials: 0.58%; mean reaction time before data cleaning: 4.62 ± 0.20 s). For each participant, only faces with evaluations of trustworthiness, dominance and threat given with a reaction time superior to 200 ms were analysed. No participant was excluded. Analyses were performed on at least 75% of the faces for all the participants (*N* = 60).

#### 2.1.4. Analyses

To investigate the effect of social motivation on threat evaluations, we ran a mixed linear regression on threat ratings, taking social motivation, ratings of dominance and ratings of trustworthiness as predictors and participants’ ID as a random factor. Following Todorov et al. [23], this model included linear and quadratic interaction effects of perceived trustworthiness and perceived dominance as well as interaction terms between these two factors. Importantly, this modelling approach allowed us to go beyond the analysis of overall ratings and to tease apart the respective weight of perceived dominance and trustworthiness.

In order to make the regression coefficients directly understandable, all the ratings were centred around the middle of the scale (5), such that a positive rating of trustworthiness corresponds to the perception of the face as trustworthy and a negative rating to a perception of the face as untrustworthy. No other transformation was applied to the ratings. Therefore, the regression coefficients can be interpreted as the increase in threat ratings for a change of one point in perceived trustworthiness or dominance. Social anhedonia scores were reverse-coded to reflect participants’ social motivation, such that high scores correspond to a higher motivation for social bonding. In addition, the scores were transformed into z-scores to facilitate the interpretation of the regression coefficients. The regression coefficients associated with social motivation thus correspond to the change in threat evaluations for a change in social motivation of one standard deviation.

The p-values reported for the social motivation effects are exact p-values, obtained via a permutation test ran over 1000 random reassignments of the social motivation scores.

### 2.2. Results and discussion

As can be seen in Fig 1B, faces perceived as less trustworthy and more dominant were rated as more threatening (*b*_*T*_ = -0.36 ± 0.04, *z* = -17.38, *p* < .001; *b*_*D*_ = 0.35 ± 0.04, *z* = 18.74, *p* < .001; Fig 1B), which replicates previous findings obtained by Oosterhof & Todorov [10]. In addition, a quadratic effect of dominance indicated that threat evaluations were more sensitive to higher than to lower levels of dominance (*b*_*D^2*_ = 0.05 ± 0.02, *z* = 6.06, *p* < .001; Statistical details of the mixed linear model are reported in S1 Table).

In line with the hypothesis of a targeted effect of the motivation for social bonding on face evaluations, there was a negative three-way interaction between social motivation, trustworthiness and dominance. The interaction indicates that compared to participants with lower social motivation, participants with higher social motivation scores granted more importance to perceived trustworthiness when evaluating threat in faces they perceived as dominant (*b*_T*D*SocMot_ = -0.02 ± 0.02, *z* = -2.97, *p* = .027; Fig 1B). More precisely, an increase in the trustworthiness rating of dominant faces resulted in a larger decrease in threat ratings for more socially motivated participants than for less socially motivated participants. However, contrary to the hypothesis of an indiscriminate increase in participants’ willingness to approach others, no main effect of social motivation was found (b_SocMot_ = -0.01 ± 0.24, *z* = -0.06, *p* > .250; no other significant effect of social motivation was found: all *p*s >.154). Although correlational, this result confirms the hypothesis that social bonding is associated with a specific increase in the importance granted to perceived trustworthiness when producing social evaluations.

## 3. Study 2 –Threat evaluations (replication)

In order to confirm this finding and to check its robustness, we conducted an exact replication of this experiment as recommended by the Open Science Framework [24].

### 3.1. Material and methods

#### 3.1.1. Participants

The same sample size as in Study 1 was used for this replication study. 60 American participants (26 women), aged 22 to 62 years old (34.83 ± 2.58 years), were recruited via Amazon Mechanical Turk (MTurk, http://www.mturk.com). As in the princeps experiment, each participant received 2$ for completing this task.

#### 3.1.2. Experimental procedure

The same experimental procedure as in the previous study was applied for this online experiment and participants completed the Social Anhedonia Scale after having completed the face evaluation task (*M* = 14.58 ± 2.27, Cronbach's *α* = .75). As in Study 1, the experiment lasted approximatively 20 minutes.

#### 3.1.3. Data cleaning and analyses

As in Study 1, trials with reaction times inferior to 200 ms were discarded for reflecting an incomplete processing of the face (percentage of excluded trials: 0.08%; mean reaction time before data cleaning: 3.76 ± 0.23 s). Analyses were performed on at least 95% of the faces for all the participants (*N* = 60). The exact same analyses as in the previous study were conducted to measure the influence of the motivation for social bonding on social preferences. As in Study 1, ratings were centred around the middle of the scale and motivation for social bonding scores were reverse-coded and transformed into z-scores.

### 3.2. Results and discussion

Confirming Study 1 results, trustworthiness decreased and dominance increased perceived threat (*b*_*T*_ = -0.28 ± 0.04, *z* = -14.09, *p* < .001; *b*_*D*_ = 0.26 ± 0.04, *z* = 13.90, *p* < .001). Crucially, the effect of social motivation on social preferences was replicated in this experiment: participants with higher levels of social motivation granted more weight to trustworthiness when evaluating threat in dominant faces compared to participants with lower levels of social motivation (*b*_T*D*SocMot_ = -0.03 ± 0.02, *z* = -3.36, *p* = .018). However, in this experiment, the main effect of social motivation on threat evaluations was significant, suggesting that social motivation was also associated with a general increase of the willingness to approach others in this sample (*b*_*SocMot*_ = -0.41 ± 0.31, *z* = -2.65, *p* = .008).

## 4. Study 3—Threat evaluations in the lab

We further tested the robustness of this finding, by replicating the same experiment on 30 participants tested in the lab, using avatar faces controlled for dominance and trustworthiness to control for participants’ ability to detect physical characteristics that are commonly associated with cooperation- and power-related impressions [25].

### 4.1. Material and methods

#### 4.1.1. Participants

30 French participants (18 women), aged 18 to 35 years old (25.43 ± 0.10 years) were recruited via an ad posted on a university mailing list. The number of participants was reduced for this study because of material constraints. However, this number is still larger that the minimal number of participants requested to evidence correlational effects of social motivation [18] (n = 26). Participants received 10€ for completing this study, which is the standard payment for studies conducted at the École Normale Supérieure.

#### 4.1.2. Procedure

The same experiment as in Studies 1 and 2 was presented using E-Prime except that participants had to evaluate realistic avatar faces instead of photographs. More precisely, we presented 80 faces generated with FaceGen 3.1 coming from 20 maximally distinct source identities were randomly selected from the face database available on Todorov’s website (http://tlab.princeton.edu). Four variations of each identity were used (extremely dominant, extremely submissive, extremely trustworthy and extremely untrustworthy; Oosterhof & Todorov, [10]), which enabled us to control for participants’ ability to recognize faces from these four categories. These avatars were automatically generated by Oosterhof and Todorov [10] based on the evaluation of the faces from Karolinska database by more than 300 participants. Importantly, multiple studies from different research teams have shown that these avatars can reliably be used to evoke different traits. As the sample size for this study was divided by two compared to Studies 1 and 2, the number of faces was doubled in this experiment to obtain the same total number of trials as in the previous studies.

As in Studies 1 and 2, participants had to rate each face on trustworthiness, dominance and threat and completed a French version of the Social Anhedonia Scale at the end of the experiment [20] (*M* = 9.60 ± 2.29, Cronbach's *α* = .84). Because we took advantage of an ongoing study, a memorization task using the same faces preceded the evaluation experiment. The experiment lasted approximately 20 minutes.

#### 4.1.3. Data cleaning and analyses

As in Studies 1 and 2, trials with a reaction time inferior to 200 ms were discarded (percentage of excluded trials: 0.04%; mean reaction time before data cleaning: 2.53 ± 0.05 s). After data cleaning, all participants had analysable responses on at least 95% of the faces. Following the same procedure as in Studies 1 and 2, ratings were centred around the middle of the scale, motivation for social bonding scores were reverse-coded and transformed into z-scores, and the data were analysed using the same linear mixed model as in Studies 1 and 2.

### 4.2. Results and discussion

As in Studies 1 and 2, perceived trustworthiness decreased perceived threat (*b*_*T*_ = -0.49 ± 0.04, *z* = -26.72, *p* < .001) and perceived dominance increased perceived threat (*b*_*D*_ = 0.43 ± 0.03, *z* = 25.83, *p* < .001). In line with the hypothesis of a specific influence of social motivation on approach behaviour, we confirmed that there was an association between social motivation and an increased weight granted to perceived trustworthiness to evaluate threat in dominant faces (*b*_T*D*SocMot_ = -0.03 ± 0.02, *z* = -4.27, *p* = .009). This experiment also revealed the absence of a significant main effect of social motivation (*b*_*SocMot*_ = 0.01 ± 0.28, *z* = 0.10, *p* > .250).

## 5. Study 4 –Likeability evaluations

We further tested the robustness of our findings by extending our results to likeability evaluations (60 participants). This study allows us to test whether the evidenced effect of social motivation generalizes to cases where participants evaluate a positive trait. More precisely, if social motivation is reliably associated with differences in social evaluations, it should not only influence avoidance-related evaluations but also approach-related evaluations.

### 5.1. Material and methods

#### 5.1.1. Participants

The same sample size as in Studies 1 and 2 was used for this replication study. 60 American participants (25 women), aged 19 to 66 years old (34.52 ± 2.63 years), participated in this online study via Amazon Mechanical Turk. As in the previous studies, each participant received 2$ for completing this 20-minute task.

#### 5.1.2. Procedure

The same experiment as in Studies 1 and 2 was presented using Qualtrics (http://www.qualtrics.com) except that the threat evaluation question was replaced by a likeability question (‘how likeable is this person?’). In order to control for participants’ sensitivity to the intensity of facial features usually interpreted as trustworthiness and dominance cues, 40 faces varying parametrically in dominance and trustworthiness generated using FaceGen 3.1 were used in this experiment [25]. Previous work has demonstrated that these faces elicit dominance and trustworthiness judgments both at the explicit and the implicit level [25,26]. As in the previous studies, participants completed the Social Anhedonia Scale (M = 15.48 ± 2.57, Cronbach's *α* = .81) after the social evaluation task. The experiment lasted approximately 20 minutes.

#### 5.1.3. Data cleaning and analyses

As in the threat evaluation studies, reaction times below 200 ms were discarded (percentage of excluded trials: 0.54%; mean reaction time before data cleaning: 5.04 ± 0.53 s). After data cleaning, all participants had analysable responses on at least 92% of the faces. As in Studies 1–3, the ratings were centred around the middle of the scale and the Social Anhedonia Questionnaire scores were reverse-coded and transformed into z-scores. The data were analysed using the same linear mixed model as in the three previous studies.

### 5.2. Results and discussion

The general pattern replicates the threat evaluation studies, with more trustworthy and less dominant faces perceived as more likeable (*b*_*T*_ = 0.40 ± 0.03, *z* = 22.63, *p* < .001; *b*_*D*_ = -0.33 ± 0.03, *z* = -21.70, *p* < .001) and likeability ratings being more sensitive to higher than to lower levels of dominance (*b*_*D^2*_ = -0.03 ± 0.01, *z* = -4.76, *p* < .001; Fig 1C).

More importantly, the effect of the motivation for social bonding on the combination of perceived trustworthiness and perceived dominance was also evidenced in likeability evaluations with highly socially motivated participants granting more weight to trustworthiness while evaluating dominant faces than less socially motivated participants (*b*_T*D*SocMot_ = 0.03 ± 0.01, *z* = 3.93, *p* = .024; Fig 1C). As in Studies 1–3, no significant main effect of social motivation was found (*b*_*SocMot*_ = 0.11 ± 0.18, *z* = 1.15, *p* = .164). This result confirms that social motivation is associated with a specific increase in the importance granted to perceived trustworthiness when evaluating the likeability of dominant individuals.

## 6. Meta-analysis of the social evaluations studies (Studies 1–4)

### 6.1. Analysis of the influence of social motivation on the importance granted on trustworthiness

As recommended by the Open Science Collaboration [27], we conducted a meta-analysis on the three-way interaction between social motivation, trustworthiness and dominance and on the main effect of social motivation across all four social evaluation studies in order to further test the robustness of our finding [28].

#### 6.1.1. Methods

The meta-analysis was conducted on the regression coefficients associated with the three-way interaction between social motivation, trustworthiness and dominance and the main effect of social motivation independently obtained in each of the four experiments. In order to have the more conservative assessment of our effect, we used a random effect model that takes into account the fact that the evidenced effect may be truly different in each of our four studies [29]. In order to analyse the models on threat ratings and on likeability ratings together, we transformed the ratings into « approachability ratings », taking likeability ratings and the opposite of the threat ratings as dependent variable.

#### 6.1.2. Results and discussion

The meta-analysis confirmed the association between high levels of social motivation and a higher weight granted to trustworthiness for the evaluation of dominant faces (*b*_T*D*SocMot_ = 0.03 ± 0.01, *z* = 7.27, *p* < .001). In addition, it confirmed the absence of a significant main effect of social motivation on face evaluations (*b*_*SocMot*_ = 0.11 ± 0.15, *z* = 1.44, *p* = .150). Therefore, it further confirmed that participants’ social motivation is robustly associated with an increase in the importance granted to cooperation-related impressions when making social evaluations. No evidence was found that participants’ motivation for social bonding is associated with an indiscriminate increase of participants’ likeability ratings.

### 6.2. Functional consequences on approachability ratings

In order to better understand the functional consequences of these weighting differences, we ran post-hoc analyses on the predictions of the meta-analytic model. More precisely, we estimated the association between social motivation and the proportion of trustworthy faces rated as approachable (i.e. either likeable or not threatening).

#### 6.2.1. Methods

Using the parameter estimates of the meta-analytic model (S2 Table), we modelled the proportion of trustworthy faces rated as approachable (dichotomized as likeable or not threatening) for each level of social motivation. In order to measure the proportion of faces perceived as approachable among those perceived as trustworthy and/or dominant, trustworthiness and dominance were dichotomized such that faces perceived as trustworthy correspond to faces with a trustworthiness rating superior to 0 and faces perceived as dominant correspond to faces with a dominance rating superior to 0.

To take into account the uncertainty of the coefficients estimates, the proportion of trustworthy faces rated as approachable (i.e. likeable or threatening) was computed for each participant using coefficients randomly drawn from normal distributions around the model’s coefficients estimates, with deviations equal to the coefficients estimates’ standard deviations. The impact of social motivation on the proportion of trustworthy faces rated as approachable was then assessed using a beta regression.

Because this simulation method may produce different results depending on the coefficients used to compute the probabilities, we repeated this procedure 100 times to ensure that our overall results would not be influenced by the characteristics of each simulation. We computed the mean effects by averaging the results of the regressions across all simulations.

#### 6.2.2. Results

Social motivation was associated with an increase in the range of trustworthy faces participants rated as approachable (*b*_*SocMot*_ = 0.57 ± 0.08, *z* = 14.55, *p* < .001). This result was driven by more positive scores for individuals who were rated as both dominant and trustworthy (*b*_*SocMot*_ = 0.69 ± 0.09, *z* = 15.35, *p* < .001; all the predicted proportions of submissive and trustworthy faces rated as approachable were equal to one, model p-value: *p* >.250; Fig 1C).

### 6.3. Discussion of the social evaluation studies (Studies 1–4)

In line with the hypothesis of a specific influence of social motivation on social evaluations, all four correlational studies revealed that a higher motivation for social bonding was associated with a larger importance granted to perceived trustworthiness when evaluating unknown faces. This finding indicates that social motivation is not associated with indiscriminate differences in social behaviour but specifically enhances the importance of social impressions that are directly linked to the probability of success during cooperative interactions.

Specifically, post-hoc analyses revealed that participants’ social motivation scores were associated with a higher probability of rating faces that were both dominant and trustworthy as approachable (i.e. likeable or not threatening). Supplementary analyses further showed that this effect was not due to basic differences in the way participants’ perceive facial features commonly used as dominance and trustworthiness cues (See Supplementary Materials). A higher motivation for social bonding is thus associated with an increase in the percentage of trustworthy faces that individuals rate as approachable. To further examine the functional consequences of this finding, we directly measured participants’ preference for dominant and trustworthy faces.

## 7. Study 5—Social preferences

The goal of this study was to estimate the association between social motivation and participants’ preference for dominant and trustworthy faces. More precisely, based on the post-hoc analysis of the social evaluation studies (section 6.2), we expect social motivation to increase participants’ preference for faces that are both dominant and trustworthy.

### 7.1. Material and methods

#### 7.1.1. Participants

The same sample size as in Studies 1, 2 and 4 was used for this study. 60 American participants (28 women) aged 19 to 67 years old (31.80 ± 2.49 years), were recruited via Amazon Mechanical Turk. Each participant received 2$ to complete this 20-minute task.

#### 7.1.2. Procedure

The experiment was programmed on Qualtrics. 16 avatar faces similar to those presented in Study 4 (generated via FaceGen 3.1 software and controlled for both trustworthiness and dominance; Todorov et al., [25]) were used in this experiment. The 16 faces corresponded to every possible combination of dominance and trustworthiness in a range of -3 to +3 points with an increment of 2 points (i.e. -3, -1, +1 and +3) [10]. The faces were regularly spaced on both the dominance and trustworthiness dimensions. In each presented pair, the faces were 2 to 6 points different from each other on at least one dimension. This resulted in 120 pairs of faces.

**Fig 2:**
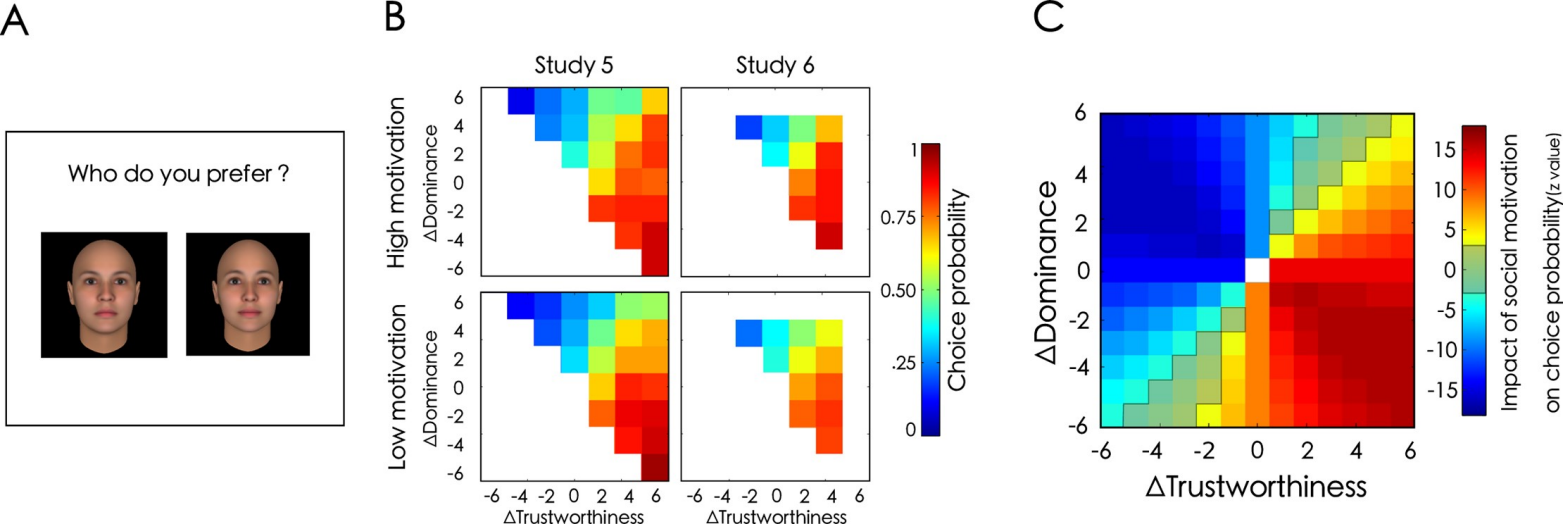
Social motivation is associated with an increased preference for dominant and trustworthy individuals. (A) Example of a preference trial. Both faces appeared simultaneously on the screen. Participants had up to 2 seconds to answer by pressing “e” to select the face on the left and “p” to select the face on the right. (B) Influence of social motivation on the average choice probability for every combination of differences in trustworthiness (x axis) and dominance (y axis) in the meta-analysis. For both Studies 4 and 5, the average probability of choosing a particular avatar within a pair is computed for each participant and compared between participants with high vs. low social motivation scores (median split). Social motivation is associated with an increased probability of preferring more trustworthy and more dominant faces. Only half of the ratings are represented since our task is perfectly symmetrical. (C) Association between social motivation and choice probability for every combination of differences in trustworthiness (x axis) and dominance (y axis) in the meta-analysis. Meta-analytic z values are represented on a scale ranging from blue for negative values to red for positive values. The use of models’ prediction allowed us to compute a preference score for each possible combination of trustworthiness and dominance. Shaded areas correspond to z-values below the 5% threshold of statistical significance after applying a Bonferroni correction for 169 comparisons. Social motivation is associated with a higher probability of preferring the most dominant and trustworthy faces.

Each trial began with a central fixation cross presented for 300 ms, then the two faces were presented simultaneously. Participants had to select their preferred face and had up to 2 seconds to answer by pressing “e” for the face on the left and “p” for the face on the right (Fig 2A). Each trial was followed by a blank page presented for about 500 ms. If they failed to answer within 2 seconds, the next trial was automatically presented. All the possible pairs of faces were presented in a random order. The presentation position (right or left) of the faces was randomized between participants. The trials were separated into three blocks of 40 trials each.

As in the previous studies, participants completed the Social Anhedonia Scale [21] (*M* = 14.27 ± 2.17, Cronbach's *α* = .87). The complete procedure lasted approximatively 20 minutes.

#### 7.1.3. Data cleaning

Two participants were removed from the analyses for pressing the same key in more than 90% of the trials. The analyses were performed on 58 participants. As in Studies 1–4, reaction times below 200 ms were discarded (percentage of excluded trials: 16.71%; mean reaction time of the included participants before filtering: 1.05 ± 0.86 s; mean percentage of analysed trials for the 58 analysed participants: 83%). Although the rate of discarded trials and participants is quite low, it is higher than in the social evaluations studies (Studies 1–4). This may be due to the fact that participants had to respond with the keyboard in the present preference study while they had to click with the mouse in evaluations studies, making it harder for them to give stereotypical responses. In addition, while the evaluation studies waited for participants’ responses to present the next trial, the next trial was automatically presented after 2 seconds in the present preference study. This feature may have made the task harder for participants and thus some participants may have miss some trials. Finally, as in Studies 1–4, the Social Anhedonia Scale scores were reverse-coded and transformed into z-scores to facilitate the interpretation of the results.

#### 7.1.4. Analyses

As recommended by McFadden [24], choices were analysed using a mixed logit logistic regression, taking subject ID, trial number and face position as random factors. As each combination of dominance and trustworthiness was presented only once, no quadratic effect of trustworthiness and dominance and no interaction between trustworthiness and dominance was added to this model, such that the probability of choosing one face in a pair was equal to 1 minus the probability of choosing the other face in the pair. Levels of trustworthiness and dominance as well as participants’ social motivation were used as regressors in the logistic model (S4 Table).

Based on these coefficients, we estimated the probability of choosing a more trustworthy but more dominant face for each level of social motivation [30]. As for the estimation of the proportion of trustworthy faces rated as approachable, we ran 100 simulations using, for each participant, coefficients randomly drawn from normal distributions around the model’s coefficients estimates, with deviations equal to the coefficients estimates’ standard deviations. For each simulation, the effect of social motivation on choice probability was assessed using a beta-regression. The presented results correspond to the coefficient estimate and the associated z-value averaged across all 100 simulations.

### 7.2. Results and discussion

This experiment confirmed that participants with a high social motivation score had a higher probability of preferring a more dominant and more trustworthy face to a less dominant and less trustworthy face (*b*_*SocMot*_ = 0.01 ± 0.00, *z* = 2.76, *p* = .001; Fig 2B). This result suggests that social motivation is associated with a specific widening of participants’ cooperation circle to include dominant and trustworthy individuals and thus, a larger range of trustworthy individuals.

## 8. Study 6—Social preferences replication

As for the social evaluation studies, we assessed the robustness of our results by conducting a replication of Study 5 involving a shorter version of the task. As in Study 5, we analysed the association between social motivation and the preference for more dominant and more trustworthy faces. Importantly, we used the same measure of social preferences and the exact same analyses as in Study 5.

### 8.1. Material and methods

#### 8.1.1. Participants

200 American participants (95 women), aged 19 to 68 years old (34.27± 1.51 years), were recruited using Amazon Mechanical Turk. This sample size was defined based on previous work on the stability of behavioural differences associated with personality traits [31]. Participants received 1.5$ for completing the 5-minute task.

#### 8.1.2. Procedure

This experiment was programmed exactly as Study 5 except that only 9 avatar faces were used, corresponding to every possible combination of dominance and trustworthiness in a range of -2 to +2 points with an increment of 2 points. In total, 36 pairs of faces were presented to the participants. The rest of the procedure was identical to that used in Study 5. As in the five previous studies, participants completed the Social Anhedonia Scale at the end of the experiment (*M* = 15.09 ± 1.50, Cronbach's *α* = .94).

#### 8.1.3. Data cleaning and analyses

Four participants were removed from the analyses for suspicion of having already completed the original preference study based on their IP address. Nine participants were removed from the analyses for pressing the same key in more than 90% of the trials. The analyses were performed on 187 participants.

As in Studies 1–5, reaction times were filtered to leave out reaction times shorter than 200 ms (percentage of excluded trials: 13.32%; mean RT before filtering: 1.05 ± 0.01 s; mean percentage of analysed trials for the 187 analysed participants: 87%). As in the previous studies, the Social Anhedonia Scale scores were reverse-coded and transformed into z-scores. The exact same analyses as in Study 5 were conducted to measure the influence of social motivation on participants’ preference for more dominant and more trustworthy faces.

### 8.2. Results and discussion

As in Study 5, participants’ motivation for social bonding was associated with a higher probability of preferring the more trustworthy and more dominant face in this replication study (*b*_*SocMot*_ = 0.01 ± 0.00, *z* = 3.18, *p* < .001; Fig 2B). This result confirms the robustness of our finding that social motivation influences people’s preferences for individuals who are both dominant and trustworthy, thereby enlarging their cooperation circles to a bigger number of trustworthy individuals.

## 9. Meta-analysis of the social preferences studies (Studies 5 & 6)

As for the social evaluation studies, we assessed the robustness of our findings by conducting a meta-analysis on these two experiments [27,28]. The aim of this final analysis was to further assess the robustness of the link between the motivation to form social bonds and the preference for more dominant and more trustworthy individuals.

### 9.1. Methods

As the meta-analysis on approachability ratings, a meta-analysis on the association between social motivation and the probability of preferring the more dominant and more trustworthy face (measured as) was conducted on the regression coefficients of the beta regressions independently obtained in Studies 5 and 6 (see Section 6).

### 9.2. Results

The meta-analysis conducted on the two social preferences experiments (Studies 5 and 6) further confirmed that a higher motivation to form social bonds is associated with a higher tendency to prefer more trustworthy and more dominant faces (*b*_*SocMot*_ = 0.01 ± 0.00, *z* = 4.16, *p* < .001; Fig 2C).

## 10. General discussion

Across six studies, we found that a higher social motivation is not associated with an indiscriminate increase individuals’ willingness to approach others but that it is instead associated with specific variations in social preferences. More specifically, we showed (Studies 1–4) that social motivation is associated with a larger importance granted to cooperation-related impressions, i.e. perceived trustworthiness, during social evaluations. Our results thus give first evidence supporting the idea that social motivation is associated with specific behavioural differences that might ultimately favour successful cooperative interactions [22].

Importantly, this weighting difference leads highly socially motivated participants to evaluate avatar faces that are both dominant and trustworthy as more approachable (i.e. more likeable and less threatening) than participants with low social motivation scores (Studies 5–6). This implies that highly socially motivated individuals see a larger range of trustworthy partners as approachable (i.e. both submissive, weakly powerful, and dominant, strongly powerful ones). Importantly, supplementary analyses confirmed that this effect cannot be accounted by basic differences in the way participants with high vs. low social motivation process facial features (see Supplementary Information). In line with our hypothesis, one potential corollary of this result is that highly socially motivated individuals maximize the number of successful cooperative activities they engage in and thereby, their fitness in cooperative environments.

The widening of participants’ cooperative circle to individuals who are both dominant and trustworthy raises the question of the value of these cooperative partners. Interactions with dominant individuals, i.e. physically and socially powerful ones [32–35], are not neutral compared to interactions with less dominant partners. More precisely, interacting with socially powerful individuals can provide immaterial benefits, such as social status [36,37], and physical power sometimes constitutes an important lever for resource acquisition [38–40]. Crucially however, cooperation with dominant individuals also brings about risks. For one thing, defection and retaliation against a strongly powerful partner is more costly than retaliation against someone who does not hold any power [41–43]. Importantly, unlike trustworthiness, dominance is not reliably associated with particular cooperative tendencies [44–48]. Interactions with dominant individuals therefore have higher stakes, but not a lower probability of success, than interactions with submissive individuals [49].

The preferences of highly socially motivated individuals for partners that are perceived as both cooperative and powerful may thus reflect a high-stake cooperation strategy. This hypothesis, taking into account both the benefits and the risks associated with wider social circles, would explain why such social preferences are not uniformly shared in the population. Importantly, this strategy may be particularly successful to obtain larger amounts of resources through cooperation. Precise investigations of this hypothesis would help to further understand the adaptive value of the motivation to form social bonds in cooperative environments. In particular, while we used a general measure of motivation for social bonding in the present paper, it would be interesting to tease apart the association between various components of social motivation (e.g., social attention, reputation management and social reward responsiveness; Chevallier, Kohls, et al., [22]). In addition, experiments testing the effect of social motivation on cooperative behavior more directly may provide further insight regarding the influence of social motivation on individuals’ social strategy (e.g., economic games measuring participants’ willingness to invest in partners varying in their probability to cooperate, in the losses they can inflict and in the gains they can provide).

At this point, it is worth emphasizing that our results are correlational and that the direction of causality should be clarified. Our assumption has been that increased social motivation impacts social evaluations and preferences but it is theoretically possible that approaching a larger range of trustworthy individuals enhances individuals’ motivation for social bonding. Indeed, positive social experiences with dominant and trustworthy individuals may increase one’s willingness to form social bonds. Similarly, specific personality traits that covary with social motivation (such as low depression scores) may mediate the link between social motivation and social preferences. The present results are thus a first step towards a better understanding of the behavioural phenotype associated with the motivation for social bonding. Further experiments investigating the influence of previous social interactions on social motivation are thus needed in order to reliably establish the causal relationship between the motivation for social bonding and cooperation strategies.

Finally, and more generally, our results raise new questions about the adaptive value of different social strategies. Indeed, while cooperation and cooperative tendencies have often been investigated as a trait (i.e. by using self-reports of trust or by analysing behaviour during economic games with unknown partners [50–52], our results suggest that measuring cooperation strategies through partner choice as a combination of potential stakes, i.e. power-related impressions, and probability of success, i.e. cooperation-related impressions, can provide new insights about individuals’ cooperative preferences.

## Supporting information

S1 File Supplementary analyses. (DOCX)

S1 Fig Distribution of the social motivation scores in each study (A) Social evaluation studies (Studies 1–4) (B) Social preference studies (Studies 5 and 6).
(PDF)

S1 Table. Mixed models statistical information (DOCX)

S2 Table Meta-analytic value of the coefficient parameters of the approachability evaluations models. (DOCX)

S3 Table Replication of the approachability evaluation models. (DOCX)

S4 Table Logistic regression coefficients obtained in the preference studies. (DOCX)
