# Supplementary material for: Social motivation is associated with increased weight granted to cooperation-related impressions in face evaluation tasks
Source: PLoS One. 2020 Apr 20;15(4):e0230011. doi: 10.1371/journal.pone.0230011 (PMC7170278; doi:10.1371/journal.pone.0230011)
Supplement: S1 File — (DOCX) [file pone.0230011.s001.docx]

**Supplementary analyses**

Facial features decoding:

In order to assess whether participants actually computed the presented stimuli, we checked whether their ratings were in accordance to the avatars’ levels of trustworthiness and dominance (Study 3 - replication study on threat evaluations in the lab - and Study 4 - study on likeability evaluations). To measure participants’ ability to decode facial features commonly associated to perceived trustworthiness and dominance, we ran mixed linear regressions on trustworthiness and dominance ratings, taking avatars’ levels of trustworthiness / dominance as regressors and participants’ ID as a random factor. As expected, intensity levels of both perceived trustworthiness and dominance were successfully perceived in the two studies (trustworthiness: all *p*s < .001; dominance: all ps < .001). In addition, highly socially motivated participants did not differ in their ability to detect these facial features (meta-analytic effect across the two studies: both *p*s > .221) and were not biased to rate faces as more trustworthy or more dominant (meta-analytic effect across the two studies: both *p*s > .250).

Reaction times:

To check that differences in perceived trustworthiness and perceived dominance combination was not due to differences in facial features processing, we measured the influence of social motivation on reaction times in the Preference studies. Reaction times were analysed to assess if social motivation affected the cognitive processes underlying the combination of social impressions. This analysis was performed using mixed linear regressions for Studies 5 and 6 on the population-level scaled reaction times, taking as regressors: social motivation, the absolute value of the difference in trustworthiness between the faces of each pair (|∆Trustworthiness| = | Trustworthiness_Left_ - Trustworthiness_Right_ |) and the absolute value of the difference in dominance between the faces of each pair (|∆Dominance| = | Dominance_Left_ - Dominance_Right_ |). This analysis revealed that social motivation was not associated with any difference in the speed at which type of social impression is processed (all *p*s > .200 for each of the two studies and for the meta-analysis across the two studies; p-values obtained via a permutation test), which suggests that the effect of social motivation was circumscribed to the way social impressions are used to produce social judgments.
