# Supplementary figures and images for: Social motivation is associated with increased weight granted to cooperation-related impressions in face evaluation tasks
Source: PLoS One. 2020 Apr 20;15(4):e0230011. doi: 10.1371/journal.pone.0230011 (PMC7170278; doi:10.1371/journal.pone.0230011)

A.

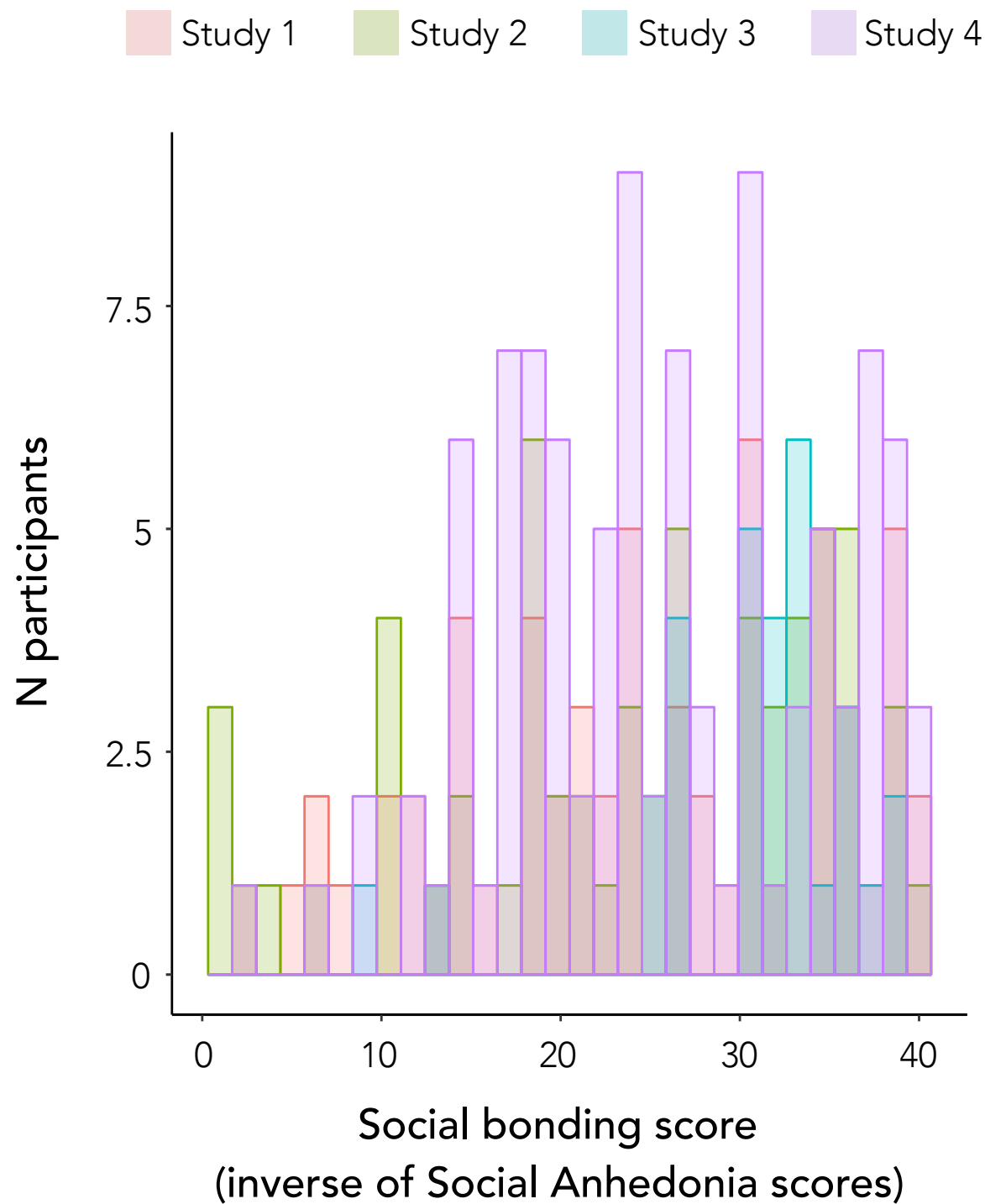

B.

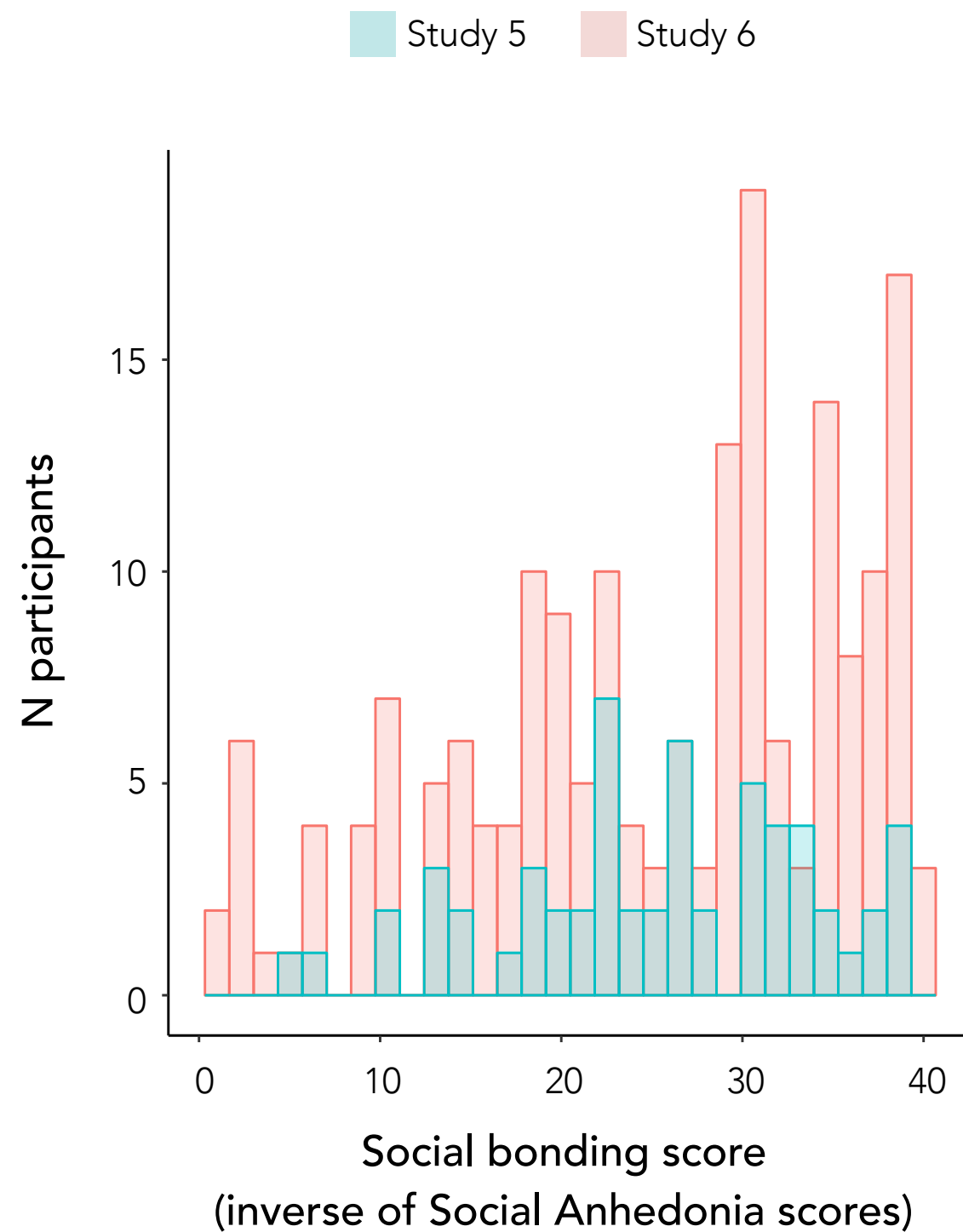

Supplement: S1 Fig — Distribution of the social motivation scores in each study (A) Social evaluation studies (Studies 1–4) (B) Social preference studies (Studies 5 and 6). (PDF) [file pone.0230011.s002.pdf]
