# Supplementary material for: Social motivation is associated with increased weight granted to cooperation-related impressions in face evaluation tasks
Source: PLoS One. 2020 Apr 20;15(4):e0230011. doi: 10.1371/journal.pone.0230011 (PMC7170278; doi:10.1371/journal.pone.0230011)
Supplement: S1 Table. Mixed models statistical information — (DOCX) [file pone.0230011.s003.docx]

**S1 Table.** Mixed models statistical information.

| **Study** | **Number of observations** | **Number of groups** | **Random effects standard deviation** | **Marginal R2** | **Conditional R2** |
| --- | --- | --- | --- | --- | --- |
| Study 1 | 2386 | ID: 60 | ID: 0.84 | 0.28 | 0.43 |
| Study 2 | 2398 | ID: 60 | ID: 1.13 | 0.19 | 0.48 |
| Study 3 | 2399 | ID:30 | ID: 0.70 | 0.47 | 0.56 |
| Study 4 | 2387 | ID: 60 | ID: 0.62 | 0.46 | 0.58 |
| Study 5 | 11594 | Trial: 5797  ID: 58  Position: 2 | Trial: 0  ID: 0  Position: 0 | 0.16 | 0.16 |
| Study 6 | 11670 | Trial: 5835  ID:187  Position: 2 | Trial: 0  ID: 0  Position: 0.03 | 0.14 | 0.14 |

ID: participant ID, Trial: combination of participant ID and trial number, Position: position of the face on the screen (right or left)
