# Supplementary material for: Social motivation is associated with increased weight granted to cooperation-related impressions in face evaluation tasks
Source: PLoS One. 2020 Apr 20;15(4):e0230011. doi: 10.1371/journal.pone.0230011 (PMC7170278; doi:10.1371/journal.pone.0230011)
Supplement: S2 Table — (DOCX) [file pone.0230011.s004.docx]

**S2 Table.** Meta-analytic value of the coefficient parameters of the approachability evaluations models.

| **Model parameters** | **Coefficients estimates** |
| --- | --- |
| Intercept | 0.78 ± 0.27 |
| Trustworthiness | 0.38 ± 0.09 |
| Dominance | -0.34 ± 0.06 |
| Trustworthiness^2^ | -0.02 ± 0.02 |
| Domininance^2^ | -0.02 ± 0.02 |
| Social Motivation | 0.11 ± 0.15 |
| Trustworthiness:Dominance | 0.01 ± 0.02 |
| Trustworthiness:Social Motivation | -0.01 ± 0.04 |
| Dominance:Social Motivation | -0.01 ± 0.05 |
| Trustworthiness^2^:Social Motivation | 0.01 ± 0.01 |
| Dominance^2^:Social Motivation | 0.01 ± 0.01 |
| Trustworthiness:Dominance:Social Motivation | 0.03 ± 0.01 |

*Meta-analytic value of the regression coefficients and their 95% confidence interval.*
