# Supplementary material for: Social motivation is associated with increased weight granted to cooperation-related impressions in face evaluation tasks
Source: PLoS One. 2020 Apr 20;15(4):e0230011. doi: 10.1371/journal.pone.0230011 (PMC7170278; doi:10.1371/journal.pone.0230011)
Supplement: S3 Table — (DOCX) [file pone.0230011.s005.docx]

**S3 Table.** Replication of the approachability evaluation models.

|  | Study 1 –  Threat evaluations | Study 2 – Threat evaluations (replication) | Study 3 – Threat evaluations in the lab | Study 4 – Likeability evaluations |
| --- | --- | --- | --- | --- |
| Study 1 – Threat evaluations |  |  |  |  |
| Study 2 – Threat evaluations (replication) | *r* = .86  *t*(10) = 5.42  *p* < .001 |  |  |  |
| Study 3 – Threat evaluations in the lab | *r* = .85  *t*(10) = 5.42  *p* < .001 | *r* = .86  *t*(10) = 5.29  *p* < .001 |  |  |
| Study 4 – Likeability evaluations | *r* = .92  *t*(10) = 7.50  *p* < .001 | *r* = .90  *t*(10) = 6.45  *p* < .001 | *r* = .88  *t*(10) = 5.92  *p* < .001 |  |

*Regression parameters on each effect (intercept, main effects and interactions) significantly correlate across the different evaluation studies revealing the robustness of the results.*
