# Supplementary material for: Social motivation is associated with increased weight granted to cooperation-related impressions in face evaluation tasks
Source: PLoS One. 2020 Apr 20;15(4):e0230011. doi: 10.1371/journal.pone.0230011 (PMC7170278; doi:10.1371/journal.pone.0230011)
Supplement: S4 Table — (DOCX) [file pone.0230011.s006.docx]

**S4 Table.** Logistic regression coefficients obtained in the preference studies.

| **Parameter** | **Original study**  **(*N* = 58)** | **Replication study**  **(*N* = 187)** |
| --- | --- | --- |
| Trustworthiness | 0.30 ± 0.02 | 0.40 ± 0.02 |
| Dominance | -0.18 ± 0.02 | -0.19 ± 0.02 |
| Trustworthiness:Social motivation | -0.01 ± 0.02 | 0.03 ± 0.02 |
| Dominance:Social motivation | 0.02 ± 0.02 | -0.02 ± 0.02 |

*Coefficient estimates of the logistic regressions used to compute the probability of choosing a more dominant and more trustworthy face and their 95% confidence interval.*
